# Glial cell-derived soluble factors increase the metastatic potential of pancreatic adenocarcinoma cells and induce epithelial-to-mesenchymal transition

**DOI:** 10.1007/s00432-023-05133-y

**Published:** 2023-08-12

**Authors:** Balbina García-Reyes, Ivan Kuzmanov, Reiner Schneider, Bianca Schneiker, Patrik Efferz, Jörg C. Kalff, Sven Wehner

**Affiliations:** 1 Department of Surgery, Medical Faculty, University Hospital Bonn, Bonn, Germany; 2 Mildred Scheel School of Oncology, Aachen Bonn Cologne Düsseldorf (MSSO ABCD), Medical Faculty, University Hospital Bonn, Bonn, Germany

**Keywords:** Pancreatic cancer, PDAC, Schwann cells, Glial cells, Cell culture models

## Abstract

**Background:**

Pancreatic ductal adenocarcinoma (PDAC) is one of the most aggressive types of cancer, characterized by the spreading of highly metastatic cancer cells, including invasion into surrounding nerves and perineural spaces. Nerves, in turn, can invade the tumor tissue and, through the secretion of neurotrophic factors, chemokines, and cytokines, contribute to PDAC progression. However, the contribution of the nerve-associated glial cells to PDAC progression is not well characterized.

**Methods:**

Two murine PDAC cell lines were cultured with the conditioned media (CM) of primary enteric glial cells or IMS32 Schwann cells (SCs). Different properties of PDAC cells, such as invasiveness, migratory capacity, and resistance to gemcitabine, were measured by RT-qPCR, microscopy, and MTT assays. Using a neuronal cell line, the observed effects were confirmed to be specific to the glial lineage.

**Results:**

Compared to the control medium, PDAC cells in the glial cell-conditioned medium showed increased invasiveness and migratory capacity. These cells showed reduced E-cadherin and increased N-cadherin and Vimentin levels, all markers of epithelial–mesenchymal transition (EMT). Primary enteric glial cell CM inhibited the proliferation of PDAC cells but preserved their viability, upregulated transcription factor *Snail*, and increased their resistance to gemcitabine. The conditioned medium generated from the IMS32 SCs produced comparable results.

**Conclusion:**

Our data suggest that glial cells can increase the metastatic potential of PDAC cells by increasing their migratory capacity and inducing epithelial-to-mesenchymal transition, a re-programming that many solid tumors use to undergo metastasis. Glial cell-conditioned medium also increased the chemoresistance of PDAC cells. These findings may have implications for future therapeutic strategies, such as targeting glial cell-derived factor signaling in PDAC.

**Supplementary Information:**

The online version contains supplementary material available at 10.1007/s00432-023-05133-y.

## Introduction

Pancreatic ductal adenocarcinoma (PDAC) is one of the most aggressive types of cancer and a leading cause of cancer-related deaths worldwide (Halbrook et al. 2023; Wang et al. 2021). It is a fatal malignancy, with a relative 5-year survival rate of only 10% in both Germany and the United States (Khalaf et al. 2021; Robert Koch Institute 2022; Rahib et al. 2014), and is predicted to become one of the leading causes of cancer-related death in the European Union in the next decades (Ilic and Ilic 2022; Lin et al. 2021). Worldwide, pancreatic cancer morbidity and mortality trends showed an upward trend in the past three decades (Ilic and Ilic 2022; Khalaf et al. 2021; Klein 2021; Lin et al. 2021), warranting novel research and therapeutic approaches.

PDAC is characterized by an abundant stroma composed of an extracellular matrix and various cell types, such as cancer-associated fibroblasts and immune cells. Nerves and their associated cell types are also observed within PDAC tumors and are considered part of the tumor microenvironment (TME) (Bunimovich et al. 2017; Cole et al. 2015; Deborde et al. 2022; Hessmann et al. 2020; Sun et al. 2022; Wang et al. 2020). Indeed, the process of perineural invasion (PNI), the spreading of highly aggressive cancer cells along and into the nerve fibers to the surrounding tissue, is a hallmark of PDAC, as it can be found in most pancreatic cancer patients (Alrawashdeh et al. 2019; Bapat et al. 2011; Felsenstein et al. 2022; Selvaggi et al. 2022). This complex crosstalk between cancer cells and nerves promotes tumorigenesis, cancer cell survival, invasiveness, and evasion of anti-tumor immune responses.

Sensory neuron ablation in a genetic PDAC model (Saloman et al. 2016a, b; Saloman et al. 2016a, b) slowed down cancer initiation and progression, indicating that nerve fibers actively support tumor growth. A molecular interaction between tumor cells and nerves has been described for an axon guidance pathway, which seems to mediate synergistic signaling between tumor cells and nerves to enhance PNI in pancreatic cancer (Jurcak et al. 2019). In contrast, less is known about the role of glial cells in PDAC biology. Glial cells in the pancreas have been identified as part of the PDAC TME (Bunimovich et al. 2017; Deborde et al. 2022; Demir et al. 2014; Sun et al. 2022, 2023). In both human tissue and murine pancreatic cancer models, Schwann cells (SCs) can be dispersed in the stroma, particularly around pre-neoplastic lesions (Demir et al. 2014). Additionally, a high level of SCs markers was associated with poor survival of patients and a higher incidence of distant metastases and vascular invasion (Su et al. 2020).

Nevertheless, the molecular crosstalk between cancer and glial cells remains poorly documented. It has been shown that pancreatic tumor cells secrete chemo-attracting factors for SCs (Ceyhan et al. 2010; Demir et al. 2016, 2017). Other components of the TME also produce factors that stimulate either PNI or SCs’ activity. For example, pancreatic stellate cells have been shown to promote PNI in pancreatic cancer through HGF/c-Met signaling (Nan et al. 2019; Zhou et al. 2012). In more recent work (Zhang et al. 2022, preprint), pancreatic tumor-associated macrophages have been found to promote the activation of SCs through the bFGF/PI3K/Akt/c-myc/GFAP pathway, and in turn, SCs promote the recruitment of macrophages through IL-33 signaling. Finally, SCs and diverse components of the tumor immune landscape are known to interact and modify the TME, influencing cancer progression (Martyn et al. 2019; Taveggia and Feltri 2022; Zhang et al. 2020).

In this study, we used two sources of glial cells to investigate the impact of glial cell-derived factors on the murine pancreatic cancer cell lines Panc02 and UN-KC-6141. First, we used our established in vitro experimental model consisting of primary murine enteric glial cells (EGCs) isolated from *muscularis externa* of the small intestine; second, the peripheral Schwann cell line IMS32. Both cell types were used instead of pancreatic SC cultures due to technical limitations in the latter’s isolation, culture, and enrichment. We found that soluble factors released by glial cells affect proliferation, survival, and chemoresistance of PDAC cells. Our data suggest that glial cells can increase the metastatic potential of PDAC cells by increasing their migratory capacity and inducing epithelial-to-mesenchymal transition (EMT), a re-programming that many solid tumors use to undergo metastasis. Conditioned media derived from either immortalized SCs or primary EGC cultures increased chemoresistance of PDAC cells.

## Materials and methods

### Cell cultures

Murine Panc02 cells (Corbett et al. 1984) and UN-KC-6141 cells, derived from the pancreatic tumor of a Kras^G12D^; Pdx1-Cre (KC) mouse (Torres et al. 2013) were used as pancreatic tumor cell models. The murine glial cell line IMS32 (Watabe et al. 1995) was used as a glial cell model. These cell lines were grown at 37 °C in a 5% CO_2_-humidified atmosphere. DMEM (Sigma-Aldrich, St. Louis, USA) supplemented with 50 U/ml Penicillin-G, 50 µg Streptomycin (Thermo Scientific), and 10% heat-inactivated fetal calf serum (Pan Biotech, Aidenbach, Germany) were used for cultures, and the cells were used for experiments when grown to confluent monolayers.

### Animals

Wild-type (wt) C57BL/6J (Janvier, Saint-Berthevin Cedex, France) were kept under specific pathogen-free (SPF) conditions in the animal housing facility of the University of Bonn (Germany). The mice were acclimatized and cohoused with littermates in groups of up to five animals for one week after transportation from the vendor. The mice were maintained under a 12-h dark/light illumination cycle at 20–25 °C and humidity of 45–65%. The mice had free access to a standard diet chow and water ad libitum. The tissue collection was performed with mice aged 8–16 weeks, weighing 20–25 g.

### Murine enteric glial cell cultures

Primary EGC cultures were prepared as previously described (Schneider et al. 2022). Briefly, EGC cultures were obtained by killing C57BL/6 mice, 8–16 weeks of age, extracting the small intestine, and cleansing it with 20 ml of oxygenated Krebs–Ringer buffer (120 mM NaCl;  5.9 mM KCl, 15.5 mM NaHCO_3_, 1.4 mM NaH_2_PO_4_, 17.5 mM Glucose, 1.2 mM MgCl_2_, 2.5 mM CaCl_2_, 100 IU/ml Penicillin, 100 IU/ml Streptomycin and 2.5 μg/ml Amphotericin). The small bowel was cut into 3–5 cm long segments and kept on oxygenated ice-cold Krebs–Ringer buffer. Each segment was then drawn onto a sterile glass pipette, and the *muscularis externa* was stripped with forceps to collect muscle tissue for further digestion steps. After centrifugation for 5 min (300 × *g*), the tissue was incubated for 15 min in 5 ml DMEM containing Protease Type 1 (0.25 mg/ml, Sigma-Aldrich) and Collagenase A (1 mg/ml, Sigma-Aldrich) in a water bath at 37 °C, 150 rpm. The enzymatic digestion was stopped by adding 5 ml DMEM containing 10% FCS (Sigma-Aldrich), and after centrifugation for 5 min at 300 × *g*, the cells were resuspended in proliferation medium (neurobasal medium with 100 IU/ml Pen, 100 μg/ml Strep, 2.5 μg/ml Amphotericin (all Thermo Scientific), FGF and EGF (both 20 ng/ml, Immunotools). Cells in proliferation media were kept for four days at 37 °C, 5% CO_2_ to promote the formation of enteric neurospheres. For experiments, enteric neurospheres were dissociated with 0.25% Trypsin (Thermo Scientific) for 5 min at 37 °C and distributed at 50% confluency on Poly-Ornithine (Sigma-Aldrich)-coated 6-well plates in differentiation medium (neurobasal medium with 100 IU/ Pen, 100 μg/ml Strep, 2.5 μg/ml Amphotericin, B27, N2 (all Thermo Scientific) and EGF (2 ng/ml, Immunotools). After seven days in the differentiation medium, mature EGCs were used for experiments or to collect their conditioned medium for further analysis.

### MTT assay

The MTT assay was used to quantify cellular metabolic activity as a measure of cell viability, using an MTT assay Kit (Abcam) according to the manufacturer’s instructions. Briefly, cells were grown in media supplements with 10% FCS, harvested using trypsin (ThermoFisher Scientific), and counted using Trypan blue and an automatic cell counter (Countess 3, ThermoFisher Scientific). Cells were plated and incubated overnight before the assay. The plates were incubated after indicated time points for three hours with MTT reagent at 37 °C. Following this incubation, cells were treated with MTT solvent for 15 min at room temperature and measured with a spectrophotometer (Tecan). Optical density (OD) was determined by measuring absorbance at 590 nm.

### Migration assay

Migration assays were performed using a Boyden chamber migration assay (Millipore) according to the manufacturer’s protocol. Briefly, cells were transferred into each Boyden chamber and incubated with the conditioned medium as a chemoattractant, which was only added to the lower chamber. At indicated time points, migrated cells were stained with crystal violet. Subsequently, the cells were imaged to determine the number of migrated cells.

### Wound-healing assay

Wound-healing assays were performed using silicone cell culture inserts (Ibidi, Germany) according to the manufacturer's instructions. Briefly, a cell suspension was added to each well in the insert. The cells were cultured until a monolayer formed and the silicon insert was removed to generate a “wound”. The cells were then monitored under a microscope to examine migration into the wound at indicated time points.

Real-time RT-PCR Total RNA was extracted at indicated time points using the RNeasy Mini Kit (Qiagen, Hilden, Germany) and treated with Deoxyribonuclease I (Ambion, Austin, TX). Complementary DNA was synthesized using the High-Capacity cDNA Reverse Transcription Kit (Applied Biosystems, Darmstadt, Germany). The expression of mRNA was quantified by real-time RT-PCR using SYBR Green PCR Master Mix (Applied Biosystems, Darmstadt, Germany). The primers are shown in Supplementary Table S1.

### Flow cytometry (FACS)

FACS analysis was performed on single-cell suspensions obtained by briefly trypsinizing the cells. The cells were resuspended in FACS Buffer (PBS and 5% FCS) and filtered using a 70-µm filter mesh. Cells were stained with either eFluor670 (manufacturer) or 7-AAD (manufacturer) according to the manufacturer’s instructions. Flow cytometry analyses were performed on FACS Canto (BD Biosciences) using FACSDiva software, and data were analyzed with FlowJo software (v10, Tree Star, Ashland, OR, USA).

### Western blotting

Cell culture samples were collected at indicated time points and lysed in RIPA buffer (ThermoFisher Scientific, Waltham, MA, USA), centrifuged at maximum speed for 20 min, and prepared with loading buffer (Biorad, Hercules, CA, USA) to load 30 µg of protein. All samples were processed using the Biorad Western Blot systems (any KD SDS-gels, Trans-Blot Turbo System) and incubated with anti-Vimentin (BioLegend 919101, San Diego, CA) overnight at 4 °C. Next, the blot was washed three times with PBS-T, incubated with HRP-secondary antibodies (ThermoFisher Scientific, Waltham, MA, USA) for two hours at room temperature, and imaged with a Biorad ChemiDoc Imaging System. β-Actin was used as a control (A5316 Sigma-Aldrich).

### Statistical analysis

Cell quantifications were performed using QuPath Version: 0.4.3 (Bankhead et al. 2017). GraphPad Prism version 9.5.1 software (GraphPad, San Diego, CA, USA) was used for statistical analysis. Unpaired t-test (two-tailed), one-way ANOVA, or two-way-comparison ANOVA tests were performed as indicated. Bonferroni corrections were performed for multiple comparisons. The significance levels were indicated as *p* ≤ 0.05 (*), *p* ≤ 0.01 (**), *p* ≤ 0.001 (***), and *p* ≤ 0.0001 (****). The data are shown as means ± standard deviation (SD).

## Results

To assess the interactions of glial and PDAC cells, we first used an in vitro model consisting of primary murine EGCs isolated from the *muscularis externa* of the small intestine. This is a well-established model in our laboratory (Ochoa-Cortes et al. 2016; Schneider et al. 2020, 2022) that was used as a replacement for primary pancreas-derived glial cells, which are technically challenging to isolate, particularly for cell culture experiments (Leven et al. 2022). Additionally, we have employed the commercially available glial cell line IMS32 (Watabe et al. 1995), a Schwann cell line from long-term cultures of adult mouse peripheral nerves. As our tumor cell models, we have employed the commonly used cell line Panc02 (Corbett et al. 1984), a model established by the direct exposure into the pancreas of C57BL/6 mice with 3-methyl-cholanthrene, and the cell line UN-KC-6141, derived from the pancreatic tumor of a Kras^G12D^; Pdx1-Cre (KC) mouse (Torres et al. 2013).

### Glial cell-derived factors inhibit proliferation but preserve viability of PDAC cells

**Fig. 1:**
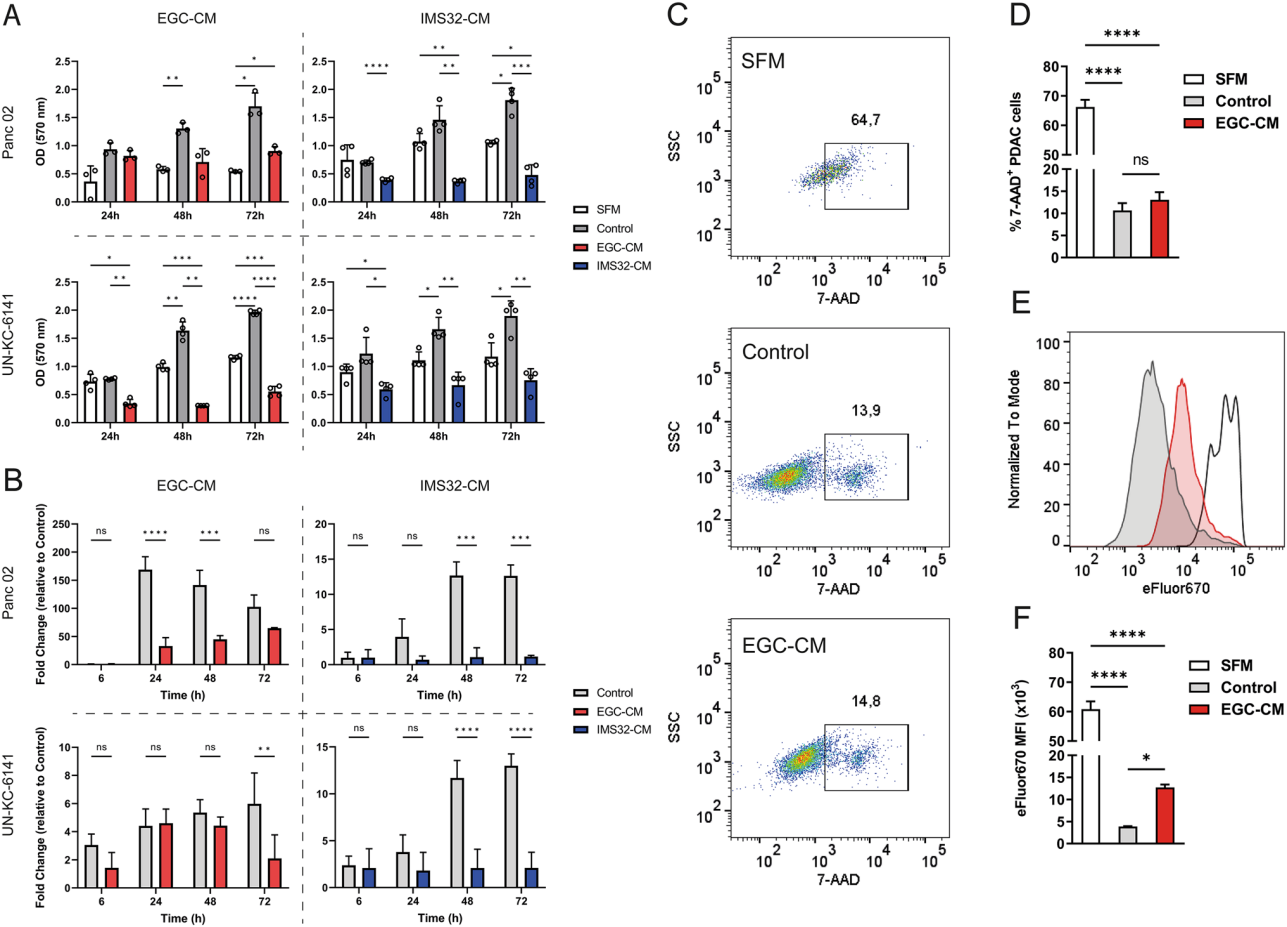
**A** The Panc02 and UN-KC-6141 cell lines were incubated with conditioned medium (CM) of IMS32 cells or EGC, unconditioned medium (Control), or SFM for 24 h, 48 h, and 72 h. Afterward, cell proliferation was determined by MTT assay (*n* = 3). Data were analyzed by two-way ANOVA with subsequent Bonferroni post hoc test. **B** The Panc02 and UN-KC-6141 cell lines were incubated with CM of IMS32 cells or EGC, unconditioned control medium, or SFM for indicated times. Afterward, the expression of the proliferation marker Ki67 was measured by RT-qPCR (*n* = 3). 18S rRNA was used as an endogenous control. Data were analyzed by two-way ANOVA with subsequent Bonferroni post hoc test. **C–D** Representative plots of flow cytometry analysis of PDAC cells after five days of culture in the presence of EGC-CM or control medium, using the dead cell exclusion dye 7-AAD (**E–F**) and cell proliferation dye eFluor670 and (*n* = 3). Data were analyzed by one-way ANOVA with subsequent Bonferroni post hoc test

We first investigated whether glial cell-derived factors alone could affect the viability and proliferation of pancreatic cancer cells. To achieve this, we performed MTT assays using murine pancreatic cancer cells treated with different glial cell-conditioned media. Panc02 and UN-KC-6141 cells were cultured in EGC-conditioned medium (EGC-CM) or IMS32-conditioned medium (IMS32-CM) and compared with their respective unconditioned medium controls (Fig. 1A). In the presence of either conditioned medium, Panc02 and UN-KC-6141 cells showed reduced proliferation as demonstrated by the reduced formazan formation in the MTT assay. Notably, we confirmed that the diminished growth was not due to the depletion of nutrients in the conditioned medium. We tested this by supplementing the conditioned medium with all nutrients commonly added to the control medium, including FCS (Supplementary Fig. S1). Only UN-KC-6141 at 48 h showed a difference in viability, and all other tested time points showed no difference between the cell viability with EGC-CM and supplemented conditioned medium (CM +).

The proliferation marker Ki67 was still detected in the treated cell lines but at lower amounts than the respective controls as determined by RT-qPCR (Fig. 1B), and no cell expansion was observed, as OD values in the MTT assay remained stable throughout the observation period for each treated cell line. Next, we confirmed that EGC-CM-cultured cells were not dying at higher rates than control cells, as shown by comparable levels 7-AAD dye, which is excluded from viable cells (Fig. 1C–D). After five days of culture, the cells cultured in serum-free medium (SFM) conditions showed, as expected, a large proportion of non-viable cells. However, no difference was observed between control cultures and cells growing in a conditioned medium. Additionally, we monitored the cell cycle of Panc02 cells under different growth conditions using the dye eFluor670. This dye is divided equally between daughter cells during each cell division (Gaté et al. 2022) (Fig. 1E–F), allowing us to compare the relative proliferation of cells under different treatments. Cells growing in serum-free conditions showed the highest intensity of eFluor670 staining, meaning fewer cells are proliferating and dividing the dye amongst daughter cells. Cells in the control growth medium had the lowest eFluor670 intensity, suggesting more cell divisions, and cell cultures in EGC-CM were at an intermediate-level staining intensity, meaning fewer cell divisions over time compared to the control. Together, our in vitro experiments showed that murine pancreatic tumor cell expansion was inhibited when cultured in a glial cell-conditioned medium while the cell viability was still preserved.

### Glial cell-derived factors increase PDAC cell resistance to the chemotherapeutic drug gemcitabine

**Fig. 2:**
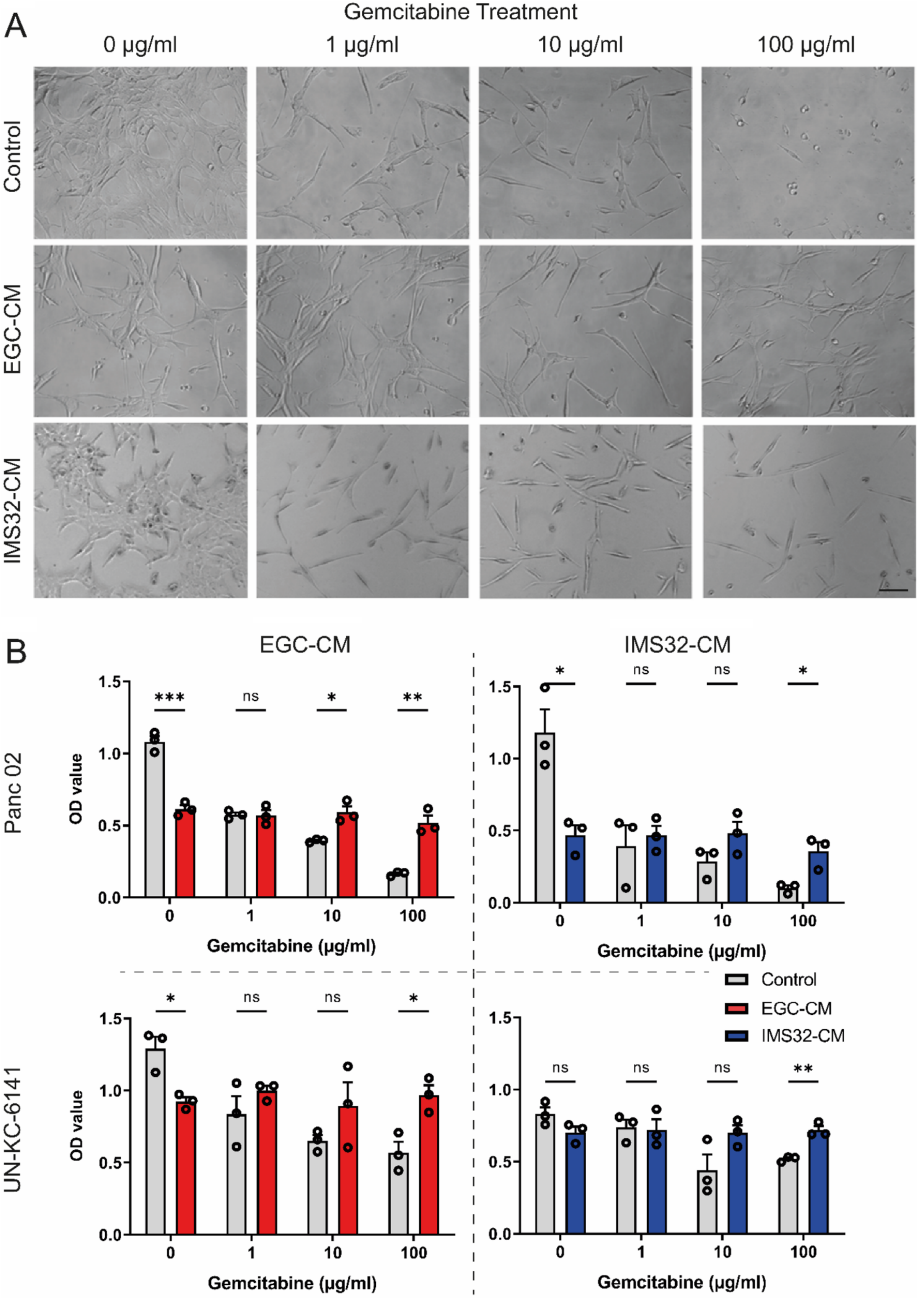
Murine PDAC cells were cultured for three days in a control medium, EGC-CM, or IMS32-CM, and in the presence of different concentrations of the chemotherapeutic drug gemcitabine. Cell morphology (**A**) was assessed by phase contrast microscopy (scale bar: 100 µm) and cell viability (**B**) by MTT assay (*n* = 3). Data were analyzed by two-way ANOVA with subsequent Bonferroni post hoc test

Gemcitabine is one of the main chemotherapy drugs used to treat pancreatic cancer and is used in all stages of the disease. However, it is common to observe the development of chemoresistance within weeks of treatment initiation (Amrutkar and Gladhaug 2017). We examined the resistance of Panc02 and UN-KC-6141 cells to gemcitabine when cultured in either a glial cell-conditioned medium or a control medium (Fig. 2). After three days of exposure to different concentrations of gemcitabine, more EGC-CM and IMS32-CM-treated cells survived at higher concentrations while also retaining their morphology, in comparison with cells growing in the control medium and exposed to the same concentrations of gemcitabine (Fig. 2A). In MTT assays, the cells cultured in both CMs resisted cell death induced by the drug. After an initial reduction in cell viability, the cells cultured in CM maintained a stable population. In contrast, the control cells exposed to increasing concentrations of gemcitabine showed a steady decrease in viability, as shown by the MTT OD values (Fig. 2B). In the case of the cell line UN-KC-6141 exposed to IMS32-CM, there was no significant reduction in cell viability despite the increased concentrations of gemcitabine. We conclude that CM from glial cell cultures has a protective effect on pancreatic cancer cell lines.

### PDAC cell invasiveness and migratory capacity are enhanced by glial, but not neuronal cell-derived factors

**Fig. 3:**
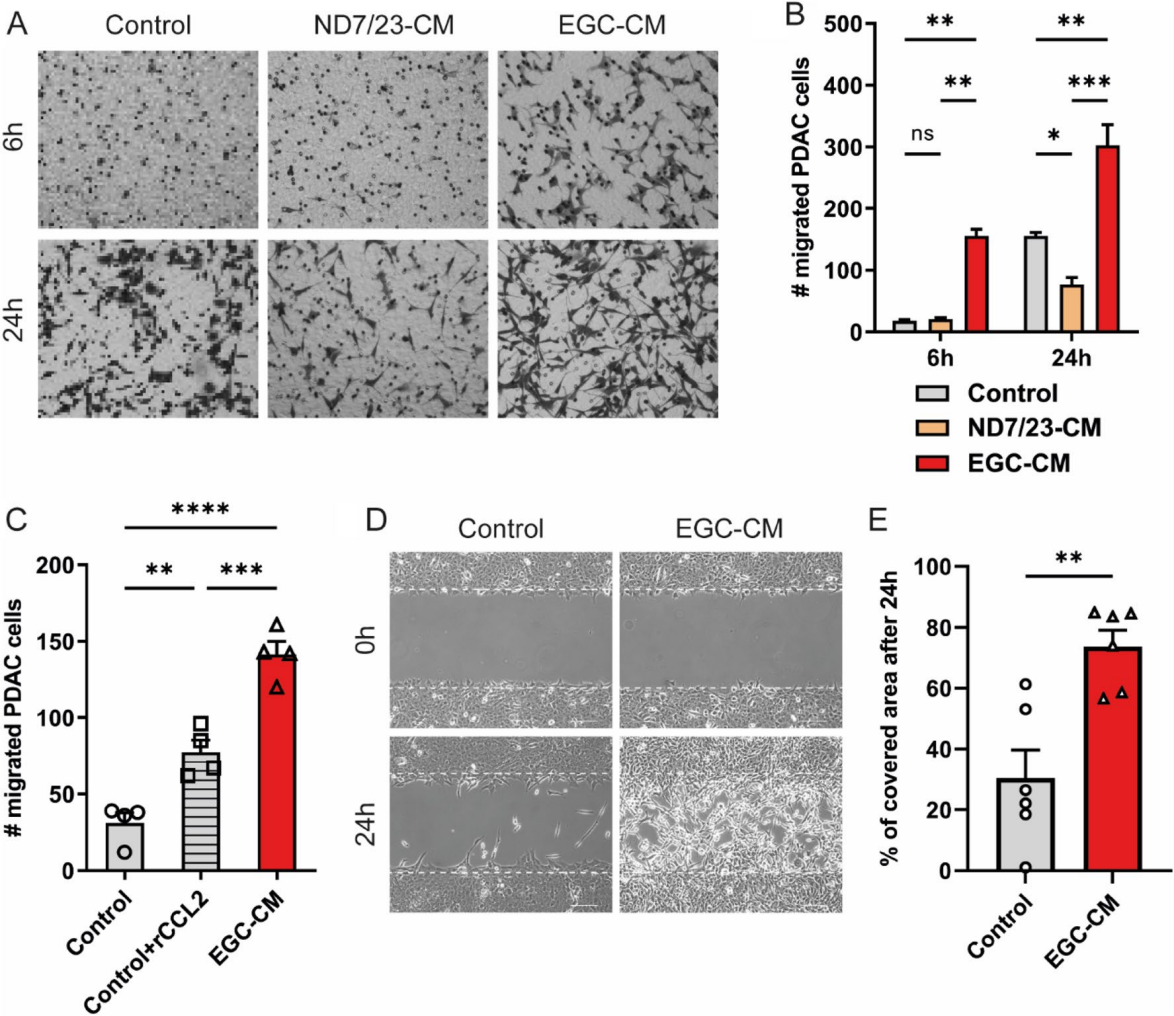
**A–B** Transmigration of PDAC cells through an 8-μm permeable insert membrane, with ND7/23-CM or EGC-CM or the control medium in lower compartments. Transmigrated cells at the bottom side of the insert membrane were **A** visualized by a crystal violet staining and **B** counted (*n* = 3). Data were analyzed by two-way ANOVA with subsequent Bonferroni post hoc test. **C** Quantification of EGC-CM triggered PDAC cell migration through an 8 µm transwell membrane after 6 h, using control medium, control medium supplemented with rCCL2 (10 ng/ml), or EGC-CM (*n* = 4). Data were analyzed by one-way ANOVA with subsequent Bonferroni post hoc test. **D** Panc02 cells were grown until confluence in a silicon barrier insert culture system. The migratory potential was assessed 24 h after removal of the silicon barrier insert and culture in the control medium or EGC-CM by phase contrast microscopy

A hallmark of PDAC cells is their strong tendency to disseminate, leading to metastasis in other organs. Thus, we analyzed if glial cell-secreted factors affect the cancer cell’s invasiveness and migratory capacity. We measured the migratory effect of these cocultures in migration assays using Boyden chambers with permeable inserts. Panc02 cells were seeded in the top of the 8-μm permeable insert in serum-free media, while EGC-CM or the conditioned medium of the neuronal cell line ND7/23 (ND7/23-CM) was added to the well below. We also included a neuronal cell line to determine which secreted factors, from either neurons or glial cells, are more relevant for migratory properties. After six hours, more Panc02 cells migrated towards EGC-CM than to non-conditioned medium or ND7/23-CM. After a twenty-four hours culture period, transmigration of Panc02 cells in the presence of the EGC-CM was still twice as high as the control medium. In contrast, ND7/23-CM inhibited migration even more than in the control medium. (Fig. 3A–B). We further investigated whether the EGC-CM effect on migration could be rescued in the control growth medium by adding CCL2, an important chemokine secreted by glial cells (Bakst et al. 2017; Schneider et al. 2022; Stoffels et al. 2014). Indeed, the addition of recombinant CCL2 to the control growth medium resulted in increased cell migration through the permeable surface. Still, the effect was not as pronounced as when using a glial cell-conditioned medium (Fig. 3C). Finally, in a wound-healing assay, EGC-CM-treated Panc02 cells repopulated a gap of defined width faster than their control growth medium counterparts did (Fig. 3D). These results indicate that the migration-stimulating effect on PDAC cells is attributable to soluble factors specifically released from the glial cell cultures and not necessarily from neurons.

### Glial cell-derived factors induce EMT in PDAC cells

**Fig. 4:**
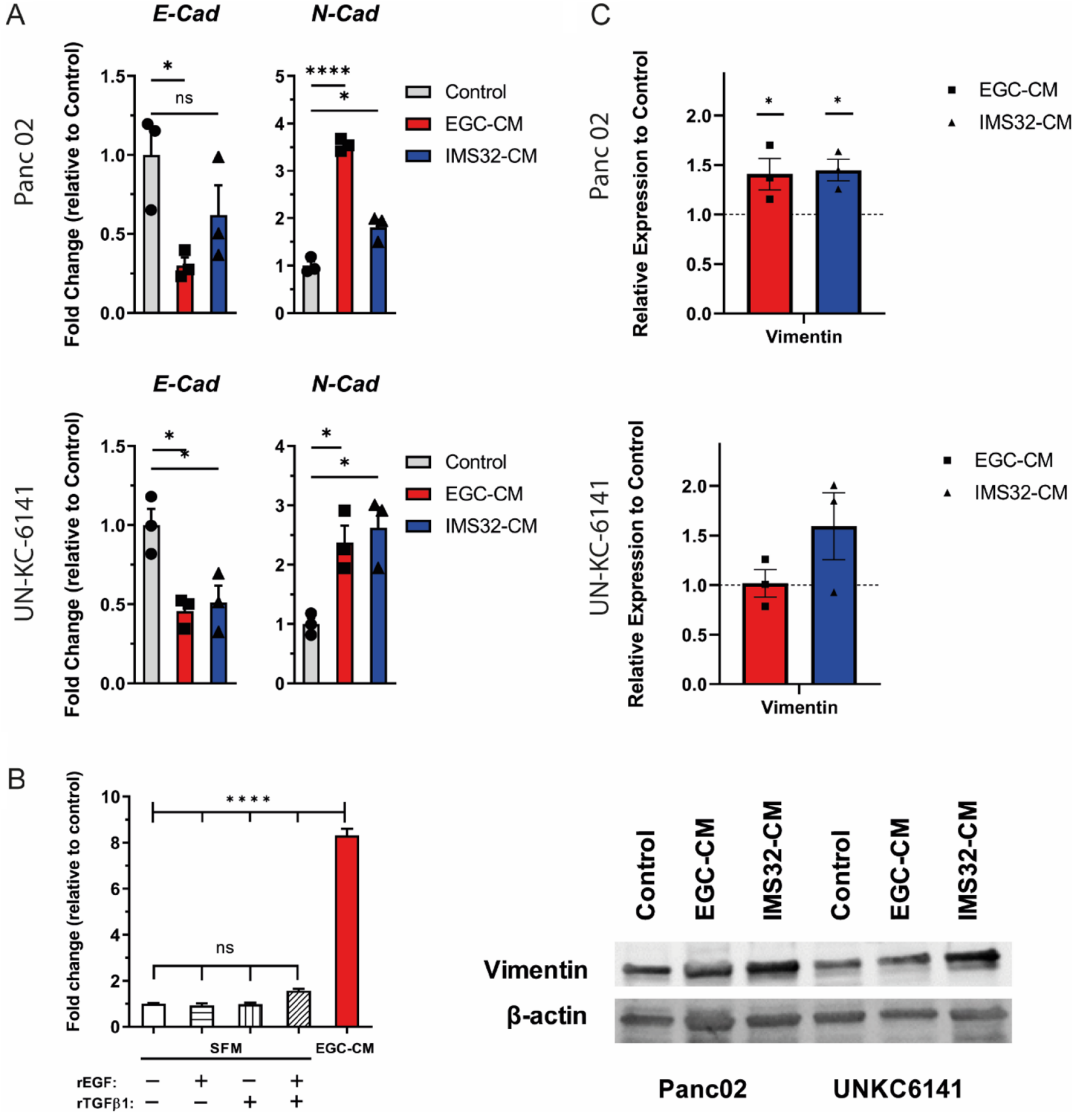
**A** RT-qPCR on Panc02 and UN-KC-6141 cells for epithelial and mesenchymal markers E-cadherin and N-cadherin, respectively, after three days in culture of EGC- or IMS32-CM (*n* = 3). Data were analyzed by two-tailed unpaired *t*-tests. **B** RT-qPCR for EMT transcription factor *SNAI1* three hours upon treatment of Panc02 cells with EGC-CM or 50 ng/ml of recombinant growth factors EGC and TGFβ1 in SFM (*n* = 3). Data were analyzed by one-way ANOVA with subsequent Bonferroni post hoc test. **C** Quantification of Vimentin protein expression compared to β-actin loading controls as determined by Western blotting in Panc02 and UN-KC-6141 cells (*n* = 3). Data were analyzed by one-way ANOVA with subsequent Bonferroni post hoc test

EMT is a fundamental process that governs normal development, but it can be hijacked by tumor cells to give rise to the dissemination of single tumor cells from the primary tumor sites (Fan et al. 2022; Lambert and Weinberg 2021). In pancreatic cancer, transcription factors associated with EMT are known to be reactivated, and EMT signature markers can be detected (Rhim et al. 2012; Rodriguez-Aznar et al. 2019). Therefore, we examined the hallmarks of EMT: downregulation of E-cadherin and upregulation of N-cadherin. Using RT-qPCR in Panc02 and UN-KC-6141 cells after culture with either control growth medium or glial cell-conditioned medium, we indeed observed downregulation of E-cadherin and upregulation of N-cadherin in cells treated with the CM in comparison with control medium (Fig. 4A). Additionally, we examined different factors that could mediate EMT in our in vitro model and found that *SNAI1* (also known as *Snail*) was upregulated in cell cultures with EGC-CM. We also tested whether the addition of factors that induce EMT, such as EGF and TGFβ, could elicit the same *SNAI1* upregulation in culture (Fig. 4B and Supplementary Fig. 2). These factors alone, or in combination, did not upregulate *SNAI1* to the same extent as treatment with EGC-CM. Finally, we examined the protein levels of vimentin (Fig. 4C), another EMT marker. We found that treatment with IMS32-CM increased the levels of Vimentin in both Panc02 and UN-KC-6141, while EGC-CM treatment only induced a slight but not statistically significant increase of vimentin in UN-KC-6141.

Overall, our results show that secreted factors from glial cells affect the properties of pancreatic tumor cells, such as proliferation, migratory capability, chemoresistance, and the EMT. These effects might enhance the tumorigenic capacity of these cells, suggesting that the interaction of glial cells and tumor cells in the TME could increase tumor development. Based on these promising data, future studies are warranted to investigate the role of glial cells in PDAC development, growth, and dissemination in vivo.

## Discussion

The peripheral nervous system can play a vital role in tumorigenesis and cancer progression, and it is considered a part of the TME (W. Wang et al. 2020). Indeed, neurogenesis and axonogenesis are present in pre-neoplastic lesions and probably contribute to early cancer initiation (Ayala et al. 2008; Mauffrey et al. 2019). There is plenty of evidence of the crosstalk between cancer cells and the peripheral nervous system (Alrawashdeh et al. 2019; Felsenstein et al. 2022; Hessmann et al. 2020; Selvaggi et al. 2022). However, most studies on this topic focus on the tumorigenic effect of nerves, while the individual impact of glial cells has been overlooked. In recent years, more evidence has been gathered on the role of glial cells in cancer development, particularly within the context of PNI (Gasparini et al. 2019; Yurteri et al. 2022). The current research literature indicates that the nervous system participates in all stages of cancer development, as well as pre-neoplastic stages (Cole et al. 2015; Faulkner et al. 2019; Kuol et al. 2018; Saloman et al. 2016a, b). However, the role of glial cells is still poorly understood, and their impact on pancreatic tumor cells is still not fully known.

Studies suggest that glial cells migrate to pancreatic tumor cells at the early stages of the disease (Demir et al. 2014); therefore, the influence of glial cells extends beyond what we know about PNI. In the present study, we demonstrated that soluble factors secreted by glial cells could enhance the tumorigenic properties of murine pancreatic tumor cells. Our study used a coculture model between two pancreatic cancer cell lines, Panc02 or UN-KC-6141, and EGCs or the SC line IMS32. We believe the EGCs can be an adequate model for studying the role of glial in pancreatic cancer-related processes. The pancreas is not only innervated by sensory, parasympathetic, and sympathetic fibers but also receives input from enteric neurons (Salvioli et al. 2002). Dye tracing experiments in rats showed that myenteric neurons from the upper duodenum project to the pancreas, terminated in the ganglia, and were observed near acini, ducts, vessels, and islet cells (Kirchgessner and Gershon 1990). In ex vivo studies using the duodenum and the attached pancreas of guinea pigs, selective stimulation of enteric neurons elicited pancreatic enzymatic secretion. When the duodenum is exposed to veratradine, it increased the metabolic activity of intrapancreatic neurons, islet cells, and acinar cells, as determined by Fos expression (Kirchgessner and Gershon 1995; Kirchgessner et al. 1994, 1996). Studies in rats have shown a neural crosstalk between the duodenum and pancreas, promoting acute pancreatitis in response to intraduodenal chemical stimulation (Li et al. 2013). Additionally, lineage-tracing experiments in mice found that Pdx1-expressing pancreas progenitors participate in the formation of enteric neurons (Brokhman et al. 2019), and enteric neural crest-derived cells are involved in pancreas development where they form neurons and SCs of intrinsic ganglia (Hutchins et al. 2018). Thus, EGCs could be used as a glial model to understand the behavior of glial cells located in the pancreas.

Our data indicate that exposure to glial cell-conditioned medium preserves the viability of pancreatic tumor cells and simultaneously reduces their ability to expand. The MTT results and eFluor670 staining show a reduction in cell expansion over time and that cultures in EGC-CM undergo fewer cell cycles over the same time compared with control growth cultures. However, our Ki67 measurement and 7-AAD staining show that cells retain their viability and do not die at a different rate than their control counterparts. These results suggest that the secreted factors from glial cells might induce the cancer cells into a more dormant or quiescent state, with growth arrest and simultaneous retention of proliferative capacity. Our findings regarding increased EMT markers in pancreatic cancer cells upon culture with glial cell-conditioned media (discussed below) are compatible with this possible quiescent state. For example, cytoskeletal changes occurring during EMT are incompatible with cell division (Datta et al. 2021).

Quiescence in cancer cells is a protective process that allows them to survive in adverse environments and become resistant to antiproliferative therapeutic agents. This dormant state is an essential factor in pancreatic cancer resistance to conventional therapy. Furthermore, tumor cell quiescence has been hypothesized to be the explanation behind tumor recurrence and metastasis in many types of cancers, including glioblastoma, breast cancer, prostate cancer, melanoma, and head and neck squamous cell carcinomas (HNSCC) (Atkins et al. 2021; Endo and Inoue 2019; Gao et al. 2019; Harper et al. 2016; Yeh and Ramaswamy 2015). However, the biological mechanisms are less understood. Most chemotherapeutic agents used in cancer treatment are mitotic inhibitors, antimetabolite drugs, and topoisomerase inhibitors that only exhibit a cytotoxic effect on proliferating cells (Sun et al. 2021; Yan et al. 2020). Indeed, we observed that cells cultured in EGC-CM resisted death induced by the chemotherapeutic agent gemcitabine. Our results are consistent with a quiescent slow-growing state, which preserves tumor cells from antiproliferative drugs.

We also found, in wound-healing assays, that EGC-CM cultures close the wound gap faster than the control growth cultures. Additionally, coculture with glial cells, but not with neuronal cells, increased the migratory ability of Panc02 cells. The presence of EGC-CM was sufficient to induce a higher migration of Panc02 cells through a permeable membrane. At the same time, the neuronal conditioned medium had only a very modest effect on migration. The reduced proliferation potential of the cells does not seem to interfere with their migratory potential, as it has also been observed, for example, that the downregulation of cyclin A1 in a breast cancer cell line increases migration and decreases proliferation (Lehn et al. 2010). It also induces resistance to apoptosis (Kajita et al. 2004; Karamitopoulou 2012; Vega et al. 2004), as we observed in our PDAC models. Supplementation of the medium with CCL2 alone was insufficient to replicate the conditioned medium’s effect on the tumor cell line. Indeed, glial cells do secret CCL2 and other cytokines and chemokines (Progatzky and Pachnis 2022; Progatzky et al. 2021), so a complex combination of glial-derived factors is responsible for the observed effect in cell migration.

Our data indicate that glial cell-derived factors induce the transcriptional and translational process of EMT in tumor cells, and we confirmed this by measurement of key markers of this process such as downregulation of E-cadherin, as well as upregulation of N-cadherin and vimentin. EMT is a process by which epithelial cells lose cell-to-cell junctions and acquire increased motility, which contributes to the invasion and progression of tumors of epithelial origin (Su et al. 2020). Furthermore, our results suggest that Snail1 mediates the EMT markers change rather than Twist, Zeb1, or Zeb2 (Supplementary Fig. S2). Many signaling pathways can induce EMT. The conditioned medium we produced from the glial cells contains a complex mixture of factors contributing to this process. These results validate IMS32 as a model to study peripheral glial cells and make it a potential replacement when the use of murine primary cells is limited or restricted. Nevertheless, the processes we examined have a multifactorial nature, and it may be possible that both IMS32-CM and EGC-CM can induce EMT and the other changes we observed in overlapping but not identical pathways.

Overall, our observations support the active role of glial cells in shaping of tumorigenic properties of pancreatic tumor cells. Our current research is focused on using in vivo PDAC models and human PDAC samples to better understand the role of pancreatic glial cells in PDAC development.

## Supplementary Information

Below is the link to the electronic supplementary material.

Supplementary file1 (DOCX 565 KB)

## Data Availability

The datasets generated during and/or analyzed during the current study are available from the corresponding author on reasonable request.
