# Supplementary material for: Glial cell-derived soluble factors increase the metastatic potential of pancreatic adenocarcinoma cells and induce epithelial-to-mesenchymal transition
Source: J Cancer Res Clin Oncol. 2023 Aug 12;149(15):14315–27. doi: 10.1007/s00432-023-05133-y (PMC10590291; doi:10.1007/s00432-023-05133-y)
Supplement: Supplementary file 1 — Supplementary file1 (DOCX 565 KB) [file 432_2023_5133_MOESM1_ESM.docx]

**Supplementary Table 1**: Oligonucleotide primers used in this publication

| **Gene** | **Forward** | **Reverse** |
| --- | --- | --- |
| *mKi67* | CCTGTGAGGCTGAGACATGG | CCCTCACTCTTGTCAGGGTC |
| *mE-cad* | GCTGGACCGAGAGAGTTAC | GGCACTTGACCCTGATACG |
| *mN-cad* | TGAAACGGCGGGATAAAGAG | GGCTCCACAGTATCTGGTTG |
| *mSnai1* | CTTGTGTCTGCACGACCTGT | CAGGAGAATGGCTTCTCACC |
| *mTwist* | AGCTACGCCTTCTCCGTCT | TCCTTCTCTGGAAACAATGACA |
| *mZeb1* | TGAGCACACAGGTAAGAGGCC | GGCTTTTCCCCAGAGTGCA |
| *mZeb2* | CCAGAGGAAACAAGGATTTCAG | AGGCCTGACATGTAGTCTTGTG |
| *mTjp1 (mZO1)* | CCGAAACCTGTGTATGCTCAAG | CTGGGCCTAAGTATCCCGTCTT |
| *mCldn5* | TAAGGCACGGGTAGCACTCA | GGACAACGATGTTGGCGAAC |
| *m18s rRNA* | GCAATTATTCCCCATGAACG | GGCCTCACTAAACCATCCAA |
| *mGapdh* | GGGAAGCCCATCACCATCTT | GCCTCACCCCATTTGATGTT |


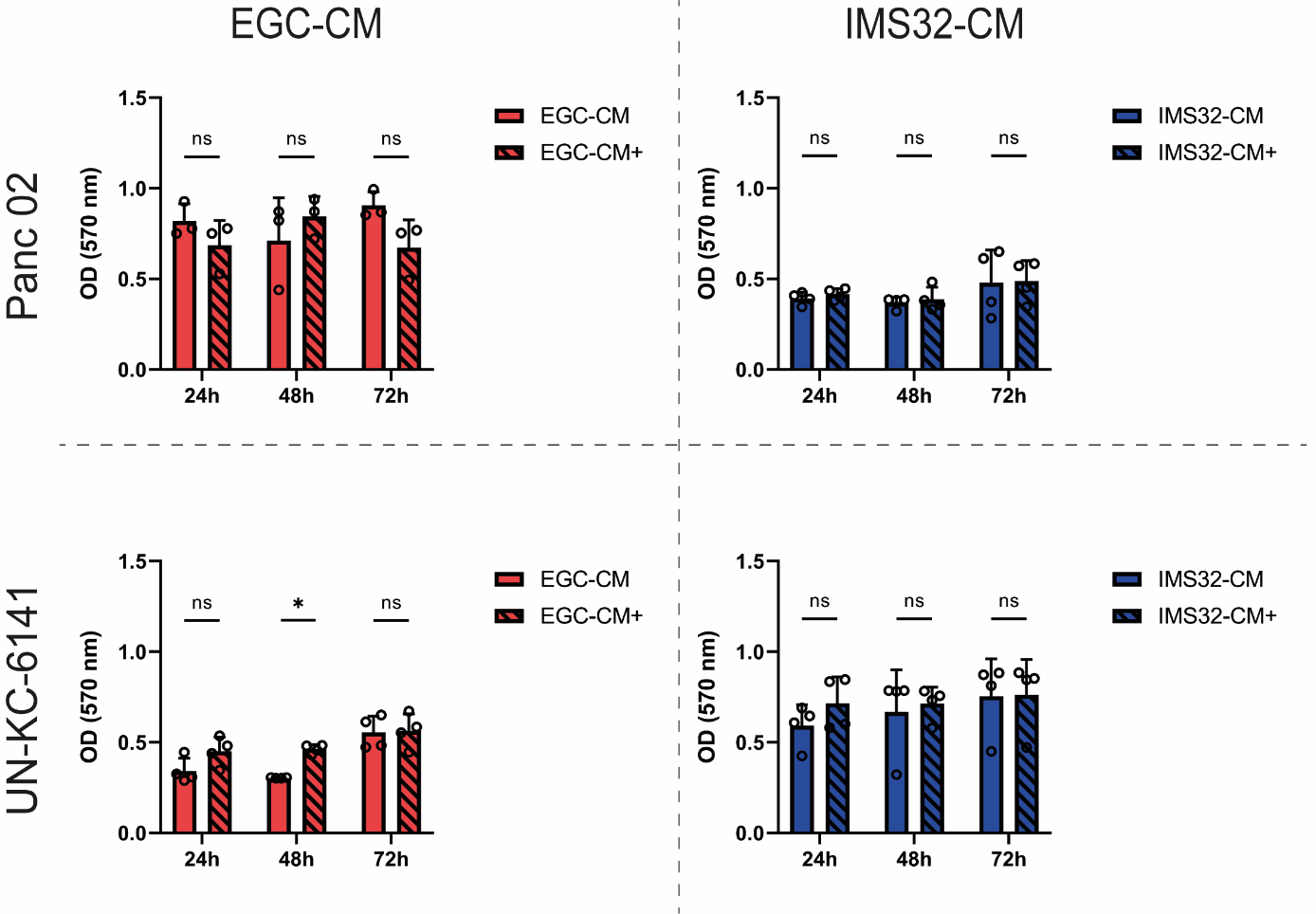


**Supplementary Figure 1**: The Panc02 and UN-KC-6141 cell lines were incubated with conditioned medium (CM) of IMS32 cells or EGC, with supplemented conditioned medium (CM+) of IMS32 cells or EGC for 24h, 48h, and 72h. Afterwards, cell proliferation was determined by MTT assay (n=3). Data were analyzed by two-way ANOVA with subsequent Bonferroni post hoc test.


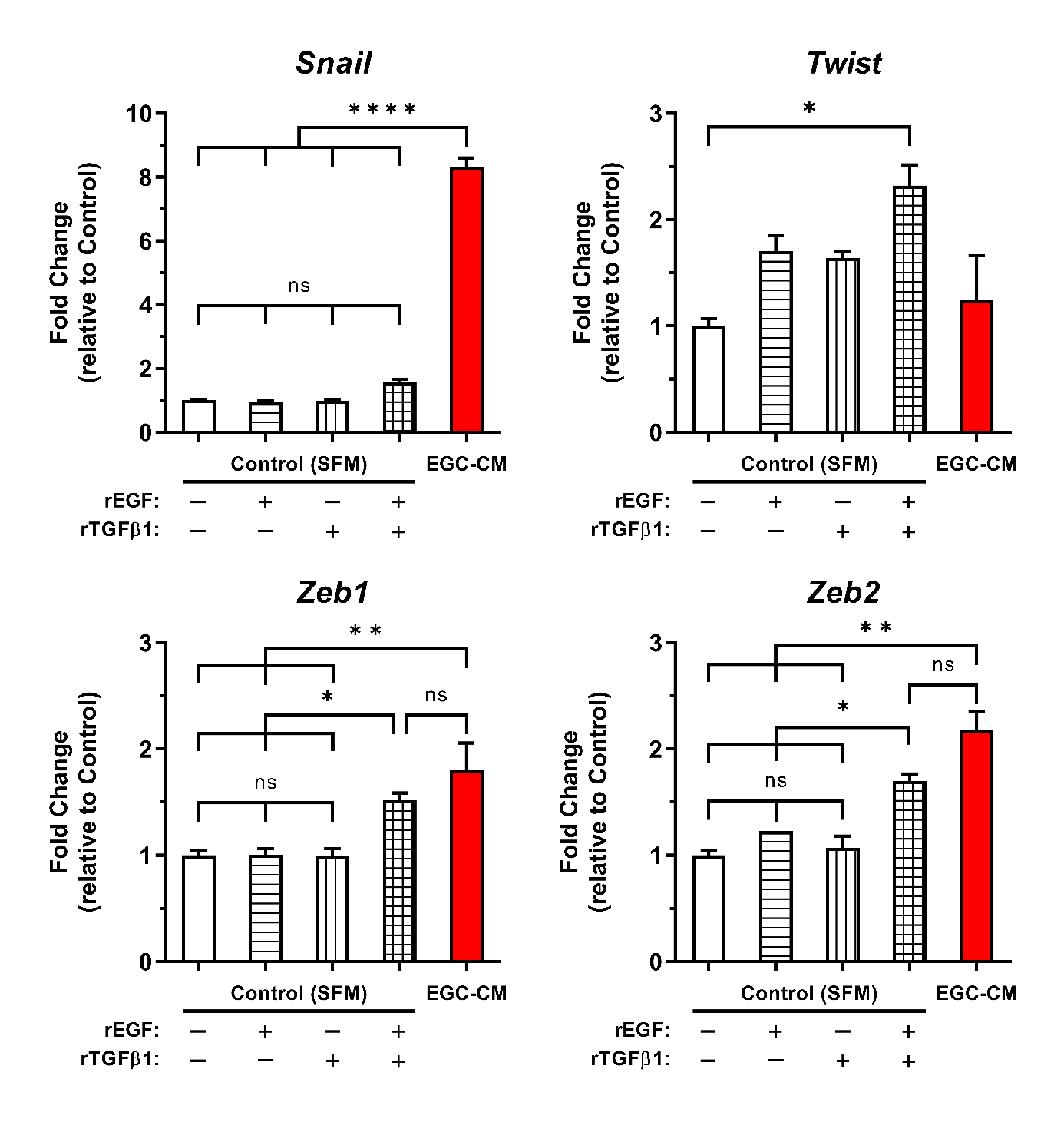


**Supplementary Figure 2**: RT-qPCR on Panc02 cells for EMT murine transcription factors *SnaiI*, *Twist*, *Zeb1*, and *Zeb2* after 3 hours of treatment with EGC-CM or 50 ng/ml of recombinant growth factors rEGC and rTGFβ1 in serum free media (SFM) (n=3). Data were analyzed by one-way ANOVA with subsequent Bonferroni post hoc test.
